# *Drosophila suzukii* energetic pathways are differently modulated by nutritional geometry in males and females

**DOI:** 10.1038/s41598-022-25509-3

**Published:** 2022-12-07

**Authors:** Sara Sario, Rafael J. Mendes, Fátima Gonçalves, Laura Torres, Conceição Santos

**Affiliations:** 1 iB2Lab – Integrative Biology and Biotechnology Laboratory, Department of Biology, Faculty of Sciences, University of Porto, Rua do Campo Alegre s/n, 4169–007 Porto, Portugal; 2 LAQV-Requimte, Faculty of Sciences, University of Porto, Rua do Campo Alegre s/n, 4169–007 Porto, Portugal; 3 CITAB - Centre for the Research and Technology of Agro-Environmental and Biological Sciences, CITAB, University of Trás-Os-Montes and Alto Douro (UTAD), Quinta de Prados, 5001-801 Vila Real, Portugal

**Keywords:** Molecular biology, Animal physiology, Entomology

## Abstract

As a polyphagous pest, *Drosophila suzukii* has a variety of host fruits available for feeding and oviposition, but how the nutritional geometry of different hosts influences its metabolism is still poorly understood. This work aimed to evaluate how *D. suzukii* metabolic and transcriptional pathways are influenced by feeding on different host fruits, and how sex influences these responses. Adult flies were allowed to feed on five different fruit-based media. Lipids, glucose, glycogen, and energy pathways-associated gene expression, were quantified. Females showed an energetic metabolism easily adaptable to the food’s nutritional characteristics; in contrast, males’ energetic metabolism was particularly influenced by food, predominantly those fed on raspberry media who showed changes in glucose levels and in the expression of genes associated with metabolic pathways, suggesting activation of gluconeogenesis and trehaloneogenesis as a result of nutritional deficiency. Here we present novel insight into how *D. suzukii*’s energetic pathways are modulated depending on fruits’ nutritional geometry and sex. While the females showed high adaptability in their energetic metabolism to the diet, males were more feeding-sensitive. These findings might be used not only to control this pest population but to better advise producers to invest in less suitable fruits based on the hosts’ nutritional geometry.

## Introduction

Diet plays an essential role in *Drosophila* overall metabolism, influencing not only their survival but also their growth, fecundity, and many other physiological and genetic traits^1–4^. In insects such as fruit flies, nutritional cues are especially relevant as the nutritional geometry of the selected food source is strongly correlated with differences in lifespan and oogenesis^5–7^. In the presence of abundant and ideal nutritional sources, flies invest energy in oogenesis and increase offspring. However, when confronted with dietary restrictions and less ideal nutrient availability, the energy reserves are shifted towards an increase in the lifespan of the adult, reducing fecundity levels and, as a consequence, a reduction in oviposition and offspring^2,7^.

The spotted-wing drosophila (SWD), *Drosophila suzukii* (Matsumura), is a polyphagous pest of soft-skinned fruits causing severe economic damage to berry producers worldwide^8^. The females prefer to lay eggs on fruits such as raspberries, blueberries, cherries, or blackberries, but eggs/larvae also feed and develop on grapes, plums, or other non-cultivated hosts^9^.

Some comparative studies have already assessed SWD oviposition preferences on different hosts^9–13^. However, available results are often unsatisfactory due to lack of reproducibility of several factors, such as cropping regimes, environmental/experimental conditions, or SWD strain in the case of laboratory assays^13^.

Increasing data show that aspects conditioning the oviposition preferences of SWD females go much beyond the conventional physicochemical characteristics of the fruits, *i.e.*, fruit firmness^14,15^, taste cues^16^, and the fruit volatile organic compounds (VOCs)^10,17–19^. Other variables, such as the microbiome profile of the host (e.g., presence of specific microbes on fruit surface) or fruit injuries, are emerging as relevant^20,21^. Hence, ranking host fruits based on female preferences is therefore complex, as those preferences appear to be influenced by more than one specific cue.

An alternative approach to overcome such complexity is to compare the potential of different fruits on SWD nutritional intake and energetic metabolism. The selection of oviposition sites by SWD females is associated with the suitability of those sites as nutritional sources for their developing offspring, as larvae are entirely dependent on those maternal choices since their development occurs exclusively inside the selected host fruit^22^. Similar to *Drosophila melanogaster,* SWD lifespan and reproduction are also closely associated with nutrient intake and availability. The nutritional geometry of the different food sources appears to have a bigger role on SWD life traits and oviposition choices than initially thought^23^. Proteins and carbohydrates are the two key macronutrients closely associated with changes in growth, fecundity, and survival in SWD^24^. When given different artificial food sources with different proteins to carbohydrates ratios (P:C), SWD flies preferred media with lower P:C, both for feeding and oviposition^23,24^. The outcome is however different when using fruit media, with flies having a higher preference and better energetic fitness in fruit-based media containing higher P:C^25,26^. Additionally, the influence of sex, previously highlighted for *D. melanogaster*^7,27^, remains unexplored for SWD. With such variable results related to preference and oviposition site selection, understanding how different hosts may influence SWD adults (and their progenies) in terms of metabolic fitness and energetic signaling pathways could give a better insight into the behavior and biology of this species, as is being proposed for *D. melanogaster*^28,29^.

When feeding on different fruits or under dietary restrictions, nutrient sensing is essential to maintaining metabolic homeostasis, especially when it comes to glucose levels^30,31^. The increase or decrease of glucose levels in the hemolymph regulates multiple metabolic pathways such as glycolysis, glycogenesis, or the polyol pathway; at the hormonal level, glucose levels are also closely associated with the insulin pathway, as the insulin-producing cells (IPC) sense glucose levels, producing *Drosophila* insulin-like peptides (Dilps) (reviewed by Mattila & Hietakangas, 2017^32^). As Dilps bind to the insulin receptor (InR) on *Drosophila* fat body cells, there is an inhibition of triacylglycerides (TAG) breakdown associated with the repression of the transcription factor forkhead box class O (FOXO), promoting TAG storage. On the other hand, the adipokinetic hormone (AKH) acts as an antagonist to insulin and promotes the mobilization and breakdown of TAG, by downstream activation of FOXO. The FOXO transcription factor is also responsible for activating the translational repressor Thor (4E-BP), which in turn is closely associated with the target of rapamycin (TOR) pathway, as well as promoting the expression of genes required for gluconeogenesis^33^. Glucose is also essential for the synthesis of trehalose, the most abundant sugar in insects’ hemolymph, and has already been reported to have an essential role in *Drosophila* survival^34,35^. Trehalose is synthesized by trehalose-6-phosphate synthase 1 (Tps1) in *Drosophila*’s fat body cells and is catabolized by trehalase (Treh) into glucose in tissues^36^.

As a polyphagous pest, with such a wide range of host fruits available, it is expected that SWD overall metabolism and survival are significantly influenced by host availability throughout the different seasons. In this study, we aim to evaluate how SWD metabolic and transcriptional pathways are influenced by feeding on different hosts, and how sex influences these responses. Unveiling the conservative profile of key metabolic pathways in SWD will give an insight into how nutritional geometry might be used not only to control this pest’s population but also to better advise producers to invest in less suitable fruits or cultivars.

## Results

### Different fruit hosts have different nutritional values

**Figure 1:**
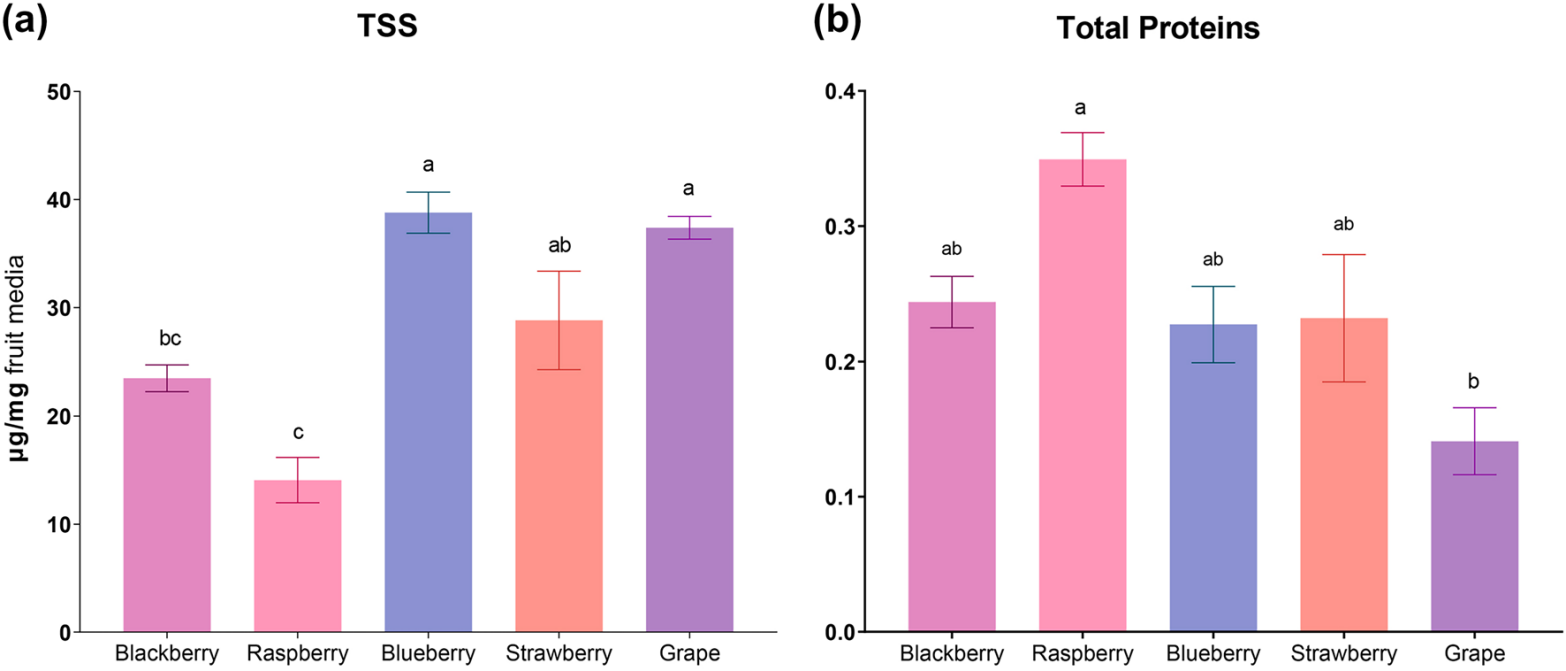
Fruit media nutritional values. (**a**) Total soluble sugars (TSS), (**b**) total proteins. Values are presented as mean ± SEM. Different letters represent statistically significant differences (p < 0.05).

To have a better insight into the different fruit media’s carbohydrate and protein contents, total soluble sugars (TSS) and total proteins were quantified in all fruit media. The raspberry medium clearly showed the lowest TSS levels compared to the other fruits, being statistically different from blueberry, strawberry, and grape media; blueberry and grape media had the highest amounts of TSS (Fig. 1a). Although blackberry-based media had intermediate levels of TSS, no significant difference was observed compared to raspberry and strawberry. On the contrary, the highest total protein content was quantified on the raspberry media, and the lowest values were found in grape media, with a statistically significant difference when comparing both, while the other media had intermediate and similar levels (Fig. 1b). A rough estimation of the Protein:Sugar (P:S) ratio of each fruit-based medium was calculated based on TSS and total protein levels. Raspberry media had the highest P:S (1:40), and grape had the lowest (1:267) (Table 1).

**Table 1:** Fruit media protein and sugar (P:S) content ratios*.*

Fruit media	P:S
Raspberry	1:40
Blueberry	1:71
Blackberry	1:96
Strawberry	1:124
Grape	1:267

### Lipids and carbohydrates vary with host and sex

**Figure 2:**
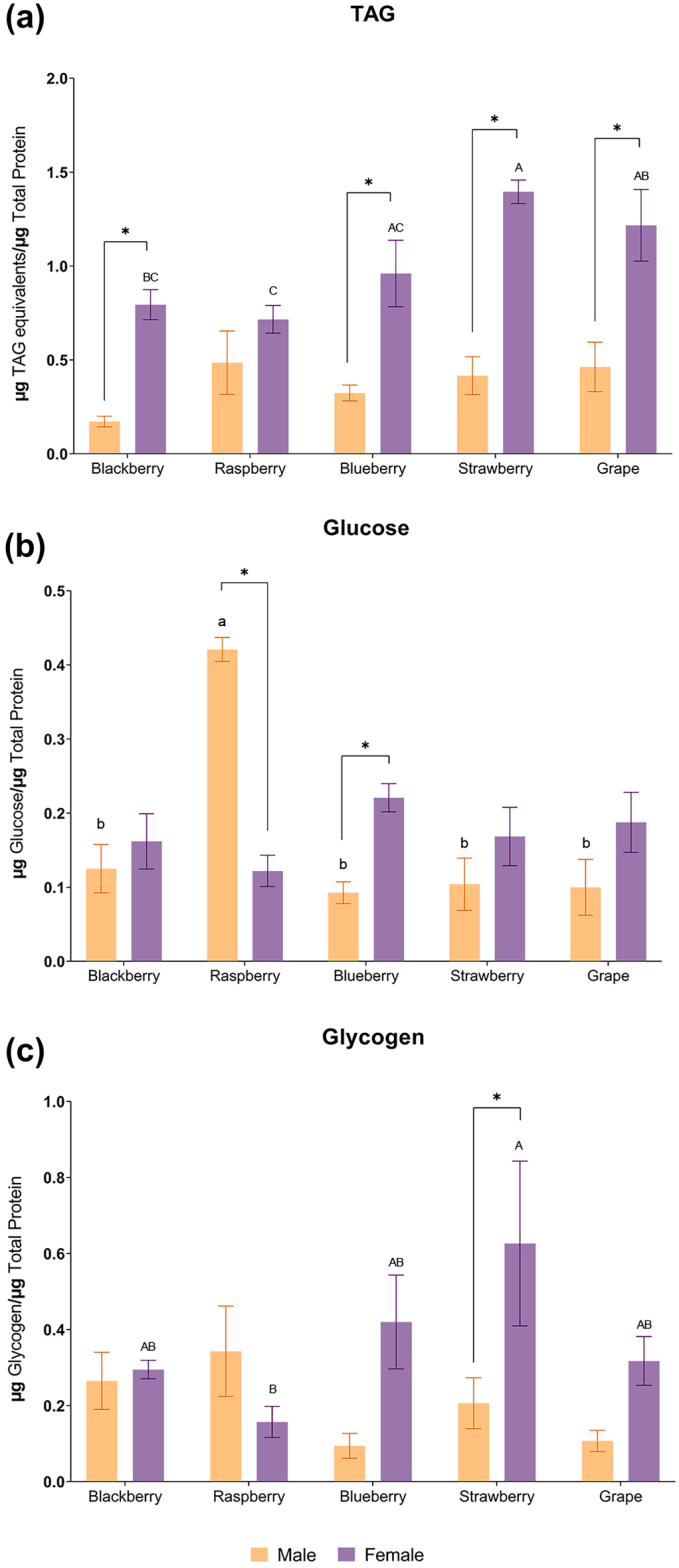
Enzymatic quantification of TAG (**a**), Glucose (**b**) and Glycogen (**c**) in adult male and female SWD. Different lowercase letters represent statistically significant differences (p < 0.05) between males, different uppercase letters represent statistically significant differences (p < 0.05) between females, and * represent statistically significant differences (p < 0.05) between males and females fed on a specific fruit media. No lowercase or uppercase letter means there was no significant difference among treatments within males or females, respectively.

Triacylglyceride (TAG) content was quantified in adult males and females to assess if there were differences in fat storage when fed on different fruit host media. Males fed on the five different fruit media had no statistically significant differences in TAG content, however, females presented statistically different TAG levels depending on the fruit medium (Fig. 2a). Females fed on strawberry medium had a significantly higher amount of TAG levels than those who fed on both blackberry and raspberry media, while female flies who fed on grape medium only had statistically significant higher TAG levels than those fed on raspberry media, which had the lowest levels of TAG. In the males, the lowest levels were found in those who fed on the blackberry medium, although with no significant difference observed between treatments. When comparing male and female flies, the levels of TAG were significantly higher in females from all media, except for raspberry.

Total body glucose and glycogen levels were also quantified. There was no difference in females’ glucose levels, however, males fed on raspberry medium had a significantly higher level of glucose than those fed on the other fruit media (Fig. 2b). Differences in glucose levels were statistically significant between male and female flies who fed on raspberry and blueberry media, with males having the highest levels after feeding on raspberry medium, and females after feeding on blueberry. Regarding the glycogen amount, females who fed on strawberry medium had the highest levels of total glycogen, while those fed on raspberry had the lowest levels, with the difference being statistically significant (Fig. 2c). All males had similar levels of glycogen, and only the females and males fed on strawberry medium had a significant difference in the glycogen levels.

### Transcript levels vary depending on the food source

**Figure 3:**
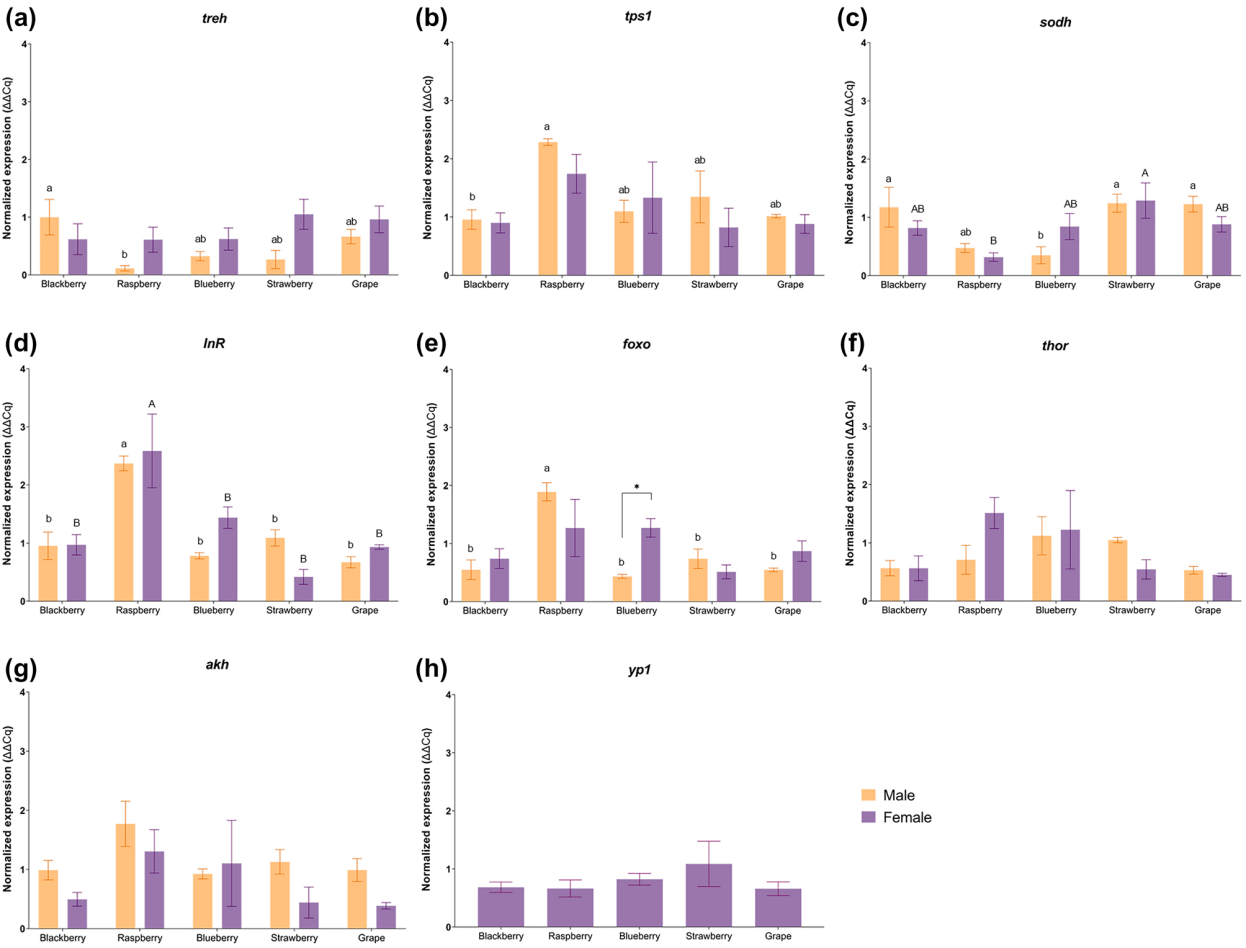
Expression of genes associated with metabolism pathways. (**a**) Trehalase (*treh*); (**b**) Trehalose-6-phosphate synthase 1 (*tps1*); (**c**) Sorbitol dehydrogenase (*sodh*); (**d**) Insulin receptor (*InR*); (**e**) Forkhead box O (*foxo*); (**f**) *thor*; (**g**) Adipokinetic hormone (*akh*); (**h**) Yolk protein (*yp1*). Different lowercase letters represent statistically significant differences (p < 0.05) between males, different uppercase letters represent statistically significant differences (p < 0.05) between females, and * represent statistically significant differences (p < 0.05) between males and females fed on a specific fruit medium. No lowercase or uppercase letter means there was no significant difference among treatments within males or females, respectively.

Transcript levels of genes associated with metabolism and energy signaling pathways were quantified by RT-qPCR. Genes associated with trehalose metabolism, trehalase (*treh*) which converts circulating and tissue trehalose into glucose, and trehalose-6-phosphate synthase 1 (*tps1*) which converts glucose into trehalose in fat body cells were differentially expressed in males; *treh* expression was significantly higher on males who fed on blackberry medium when compared to males fed on raspberry medium (Fig. 3a), while *tps1* was significantly more expressed on males fed on raspberry medium when compared to blackberry (Fig. 3b). Females had similar transcript levels of *treh* and *tps1*, with no statistically significant differences between fruit hosts. Sorbitol dehydrogenase (*sodh*, Fig. 3c), an enzyme that converts sorbitol to fructose, was affected by fruit media feeding both in males and females. Males fed on blueberry had significantly lower transcript levels of *sodh* than those who fed on the grape, blackberry, and strawberry media, while in females the lowest levels were found on those that fed on raspberry medium, which were significantly different only when compared to females fed on strawberry.

The expression of genes associated with the insulin signaling pathway, insulin receptor (*InR*), the forkhead box transcription factor (*foxo*), *thor*, the adipokinetic hormone (*akh*), and yolk protein 1 (*yp1*) was also quantified. In both males and females who fed on raspberry media, *InR* transcript levels were significantly higher than in both males and females fed on other fruit media (Fig. 3d). Males who fed on raspberry medium also had increased levels of *foxo*, being this increase statistically significant when compared to those who fed on the other media. No differences were found in the quantification of *foxo* in females, however, females fed on blueberry had a significant increase when compared to males fed on the same medium. There was no significant difference in the expression of *thor* and *akh*, both in male and female individuals, as well as in females’ *yp1* transcript levels (Fig. 3e–h).

### Male metabolism is more sensitive to the fruit host than that of females

**Figure 4:**
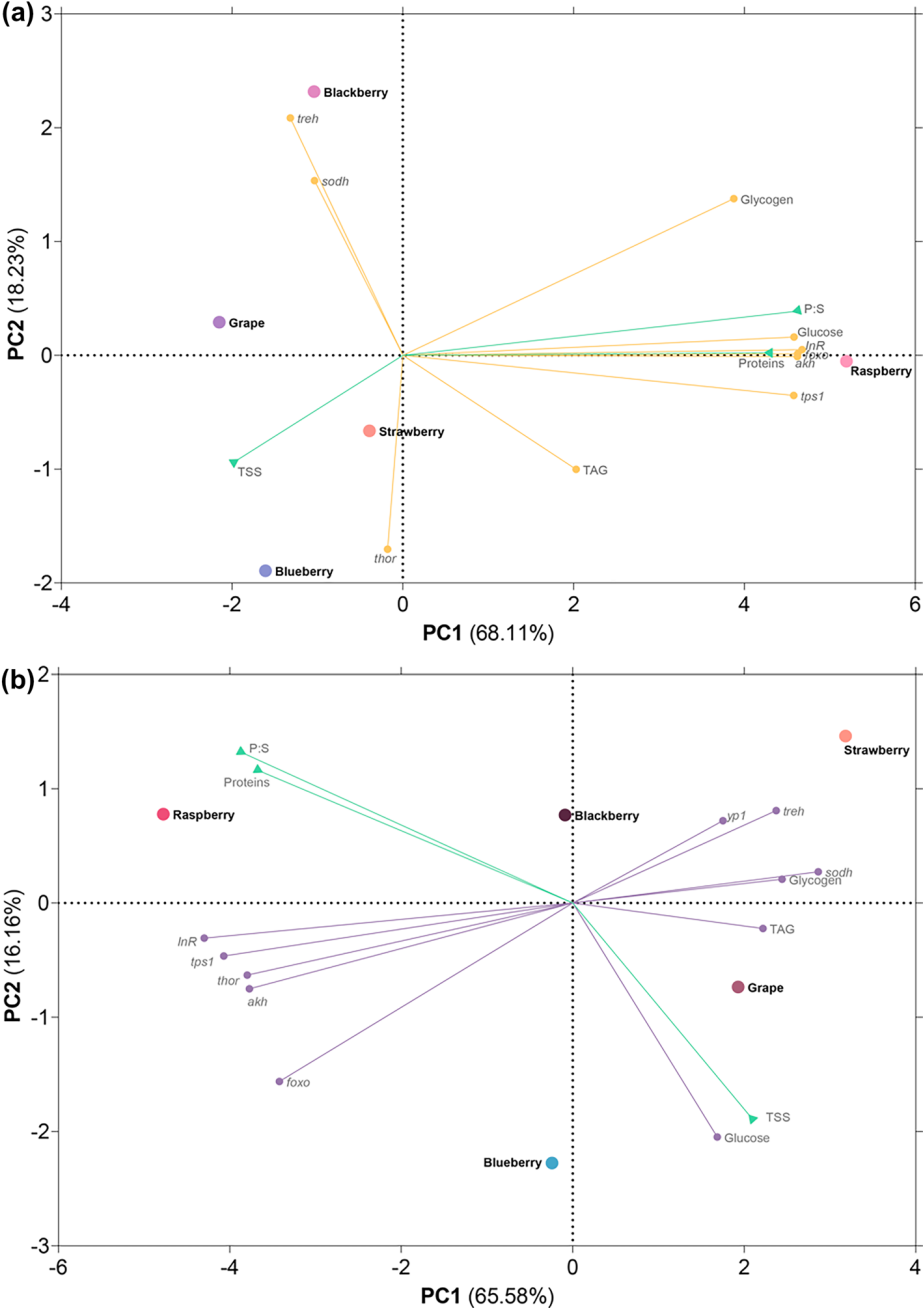
Principal component analysis of the results from fruit media analysis (green vectors) and SWD enzymatic quantification and gene expression. (**a**) results of males fed on the different fruit media (yellow vectors); (**b**) results of females fed on the different fruit media (purple vectors).

A PCA analysis was performed to better understand the correlation of the fruit-based media P:S ratio and the TSS with each media (green vectors in Fig. 4), and how the fruit-based media influence the biochemical and genetic parameters in the males (yellow vectors in Fig. 4a), and in females (purple vectors in Fig. 4b). In males, most of the analyzed metabolic parameters were heavily correlated with raspberry medium feeding. PC1 explains 68.11% of the variance and, while the majority of the fruit hosts appear on the left of PC1, raspberry clusters on the far right of PC1. Gene expression of *treh* and *sodh* strongly correlate with blackberry medium feeding. Most of the other parameters (except TSS, *thor,* and TAG) were mostly influenced by raspberry. In females, there was not a clear grouping of parameters with one specific fruit host media, however, raspberry was once again separated from the other hosts by the PC1 (which explains 65.58% of variance); parameters such as the expression of *InR*, *tps1*, *thor* and *akh* are more closely associated with raspberry medium feeding, while expression of *yp1*, *treh, sodh*, and levels of glycogen and TAG group in proximity to strawberry and grape media.

## Discussion

Polyphagous insect species, such as SWD, have a wide variety of host fruits available for feeding and oviposition. Oviposition choice by females is dependent on visual, olfactory, and social cues, with the host fruit’s nutritional geometry gaining attention as a possible factor influencing this choice. For example, it was already reported that diet plays an important role in the survival and fecundity of SWD adults and larvae^13,25^.

The metabolic and energetic signaling pathways are already well characterized in *D. melanogaster,* a well-studied model of obesity and diabetes, and are closely associated with nutrient richness such as protein:carbohydrate content^4,22^. Transferring this knowledge to SWD could contribute to understanding the influence of nutritional geometry on SWD energy and overall fecundity and survival, which modulation appears to have a key role in this pest’s survival during the colder months (overwinter)^37^, and distinguish polyphagy performances of males and females.

The SWD host fruit-based diets tested in the present study, namely, blackberry, raspberry, blueberry, strawberry, and grape, have differences in total protein and sugar content, with raspberry having the highest P:S ratio (and the lowest TSS levels) and grape having the lowest P:S ratio. Dietary proteins are known to stimulate and regulate *Drosophila* reproduction in both females (oogenesis and vitellogenesis) and males (sperm production)^5,38–41^ while carbohydrates are an essential source for glycogen synthesis and fat storage^32,42^. Previous studies already pointed to raspberry as being the most suitable host for SWD in terms of preference and best reproductive performance^13^, suggesting that SWD has a preference for high P:S fruits. However, there is still a lack of knowledge on how these fruits impact the flies’ metabolism and associated signaling pathways.

SWD females’ levels of glycogen and TAG were significantly different depending on the feeding fruit media available, with the biggest difference occurring between females who fed on raspberry medium (lowest glycogen and TAG levels) and those fed on strawberry (highest glycogen and TAG levels), while total glucose levels showed a slight trend to increase on females fed on blueberry media. TAG storage and catabolism are closely associated with oogenesis and embryogenesis, respectively. This requires a relatively high accumulation of maternal TAG levels on mature oocytes to ensure consequent embryo survival^43^. Considering that the expression of the yolk protein gene (*yp1*) was similar among the females fed on the different host media, and dietary proteins are essential for yolk protein synthesis, differences in TAG levels among females are likely due to the different carbohydrate content in the five fruit media. The highest TAG levels were found in females fed on fruit diets with higher sugar content, strawberry and grape (and therefore lower P:S ratio), and the lowest levels in females from raspberry medium, which had the lowest sugar content. This difference in TAG levels of SWD exposed to different P:S feeding media was also observed by Shu et al.^25^, who reported that females fed on raspberry (highest P:S) had significantly lower levels of TAG than those who fed on grape (lowest P:S). In *D. melanogaster*, this association was also observed, as an increase in protein availability in the feeding media led to decreased TAG levels, while a high sugar diet promoted TAG accumulation^44^. Overall, and considering the expression of metabolic pathways-associated genes, SWD females’ metabolism appears to be only slightly affected by the different nutritional geometries of the fruits, as most of the quantified gene expressions were similar among the differently fed females. This profile suggests that females rapidly shift their metabolism to adapt to the available fruits, which also explains the high reproductive success of this pest in such a wide variety of hosts.

Interestingly, males had the highest glucose levels after feeding on raspberry medium, and the lowest levels after feeding on blueberry, despite being the fruit media with the lowest and highest sugar content, respectively. The significant increase in glucose levels in raspberry-fed males could be associated with an overconsuming of sugars to regulate protein intake and maintain protein levels below a certain threshold, as proposed by Almeida de Carvalho and Mirth^45^ for *D. melanogaster.* These authors observed that *D. melanogaster* larvae overconsumed carbohydrates when developing on high protein, low carbohydrate media^45^. To further understand how sugar homeostasis is being controlled in raspberry-fed males, it is important to analyze the expression of the metabolic pathway-associated genes.

**Figure 5:**
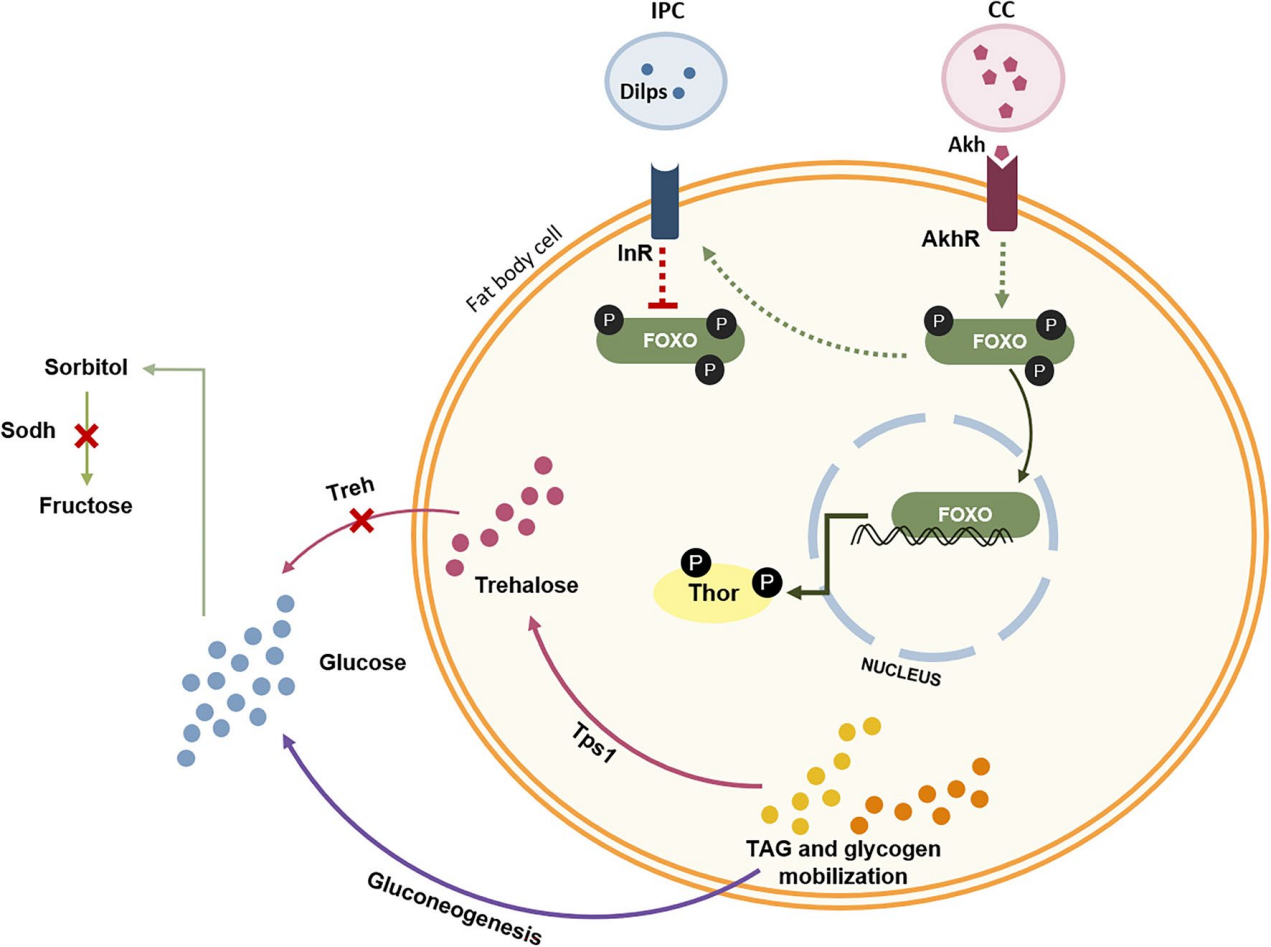
Schematic representation of the proposed hypothesis on the modulation of males’ metabolism after feeding on raspberry fruit medium. Nutrient deficiency, specifically carbohydrate deficiency, activates the production of Adipokinetic hormone (Akh) in the Corpora Cardiaca (CC) which will bind to the Akh receptor (AkhR), promoting the dephosphorylation of the Forkhead box O (FOXO) transcription factor and mobilization to the nucleus. This will promote TAG and glycogen mobilization for glucose production by gluconeogenesis, and trehalose production by trehaloneogenesis mediated by Trehalose-6-phosphate synthase 1 (Tps1). Due to low sugar content in raspberry, there is a need to increase glucose and trehalose levels, therefore the expression of Trehalase (Treh) and sorbitol dehydrogenase (Sodh) remain unchanged. Simultaneously, there is a feedback activation of the insulin receptor (InR) by FOXO, in order to maintain the receptor more prone to binding of the Drosophila insulin-like peptides (Dilps) produced by the insulin-producing cells (IPC) after the detection of higher levels of glucose.

Gene expression levels of *InR*, *foxo,* and *akh* were higher on males fed on raspberry medium. With a high expression of *InR*, we expected that the same flies would present lower expressions of *foxo*, as binding of Dilps to InR would induce suppression of FOXO. However, when looking at the expression of *akh*, we hypothesize that *foxo* is regulated by the AKH pathway, rather than being regulated by the insulin pathway. Therefore, the reduced levels of TAG on raspberry-fed flies are a consequence of its mobilization and breakdown triggered by the AKH signaling pathway. The AKH signaling pathway was already associated with gluconeogenesis and trehaloneogenesis in *D. melanogaster*, with its activation linked with nutrient deficiency, especially in low carbohydrate diets^45,46^. Also, studies on *D. melanogaster* already associated an increase in *foxo* expression with an increase in *InR* expression levels under low sugar diets, due to the feedback activation of InR by FOXO^47,48^. Thus, besides possible overconsumption of carbohydrates to counterbalance protein intake, the high glucose levels observed in males after feeding on raspberry medium could be also associated with the mobilization of energy sources to increase glucose and trehalose levels and therefore maintain sugar homeostasis. This also explains why *tps1* expression levels are higher in these males, whereas *treh* and *sodh* remain at low expression levels. The proposed hypothesis is resumed in Fig. 5. However, further studies are required to better understand how raspberries modulate SWD metabolism and could be affecting their survival and overall behavior.

When comparing males’ and females’ metabolism, the most significant differences are found in TAG levels. This occurred for males and females’ who fed on all host fruit media, except on raspberry, which is explained by the oocyte-associated TAG accumulation in females, as males do not require this “extra” TAG storage.

By analyzing glucose, glycogen, and TAG levels, as well as the expression of genes relevant to sugar metabolism and homeostasis pathways, it was possible to observe that males and females had different metabolic responses (PCA, Fig. 4). Males’ metabolism was highly sensitive to raspberry medium feeding, with most of the analyzed parameters having a strong correlation with this fruit media; on the other hand, females’ metabolism differences are not as evident, with no strong correlation with a specific fruit host. These differences in metabolic responses dependent on sex were already observed in *D. melanogaster* fed on a low sugar diet^48^, which also appears to suggest the higher plasticity of females to adapt to different nutrient availability.

The knowledge on the influence of hosts’ nutritional geometry in SWD metabolism can be applied in a host specific perspective as a control measure. Several studies already pointed to differences in susceptibility to SWD oviposition among host cultivars, such as blueberry or grape^49,50^. If the nutritional geometry of these cultivars is also taken into consideration (taking into account that different varieties have different nutritional geometries^51^), in addition to physicochemical characteristics, producers can be advised to invest in less susceptible cultivars, that provide fewer fitness advantages to SWD, and therefore reduce not only the yield losses associated with SWD infestation as well as SWD populations.

## Conclusions

SWD males and females’ metabolism was differently affected by feeding on five different host fruit-based media. While females’ metabolism is slightly affected by the differences in nutritional geometry of the different host fruit media, males’ metabolism was strongly modulated by the raspberry feeding, the fruit medium with higher protein content and lower sugar content (higher P:S). As nutritional geometry and carbohydrate/lipid metabolism play such an important role in *Drosophila* spp. survival, further studies on SWD food and oviposition preferences should also evaluate the metabolic pathways associated with nutrient sensing (as well as different feeding time points), as they could aid in better understanding this pest’s behavior and survival. Also, this correlation of oviposition cues with nutrient-dependent trade-offs and metabolic plasticity could offer better insight into more suitable management protocols, as polyphagous pest species have a higher chance of survival. This is the first report on the expression of SWD males vs. females’ energy-related pathways in response to different feeding sources varying in P:S ratios and reinforces the necessity to include both sexes in this type of studies.

## Methods

### Insect rearing

A laboratory colony of SWD was established in 2019 from infested raspberries collected in Montemor-o-Velho (40.25238882237525, − 8.688542786693263), Portugal (strain MV19). Flies were reared on a cornmeal diet [1% (w/v) agar, 4% (w/v) baker’s yeast, 8% (w/v) cornmeal, 10% (w/v) sugar, 0.15% (v/v) orthophosphoric acid and 0.08% (w/v) methylparaben] in plastic vials in an incubator at 24 ± 1 °C, 60 ± 10% RH and under a photoperiod of 16:8 (L:D) h. For every experiment, one-week-old flies were used and transferred to the respective experimental vial (10 flies per vial, 5 males and 5 females).

### Fruit host media feeding experiments

Five different fruit hosts were used to assess their effects on SWD metabolic fitness: strawberry, blueberry (‘Ochlockonee’), raspberry, blackberry, and table grape (‘Midnight Beauty’). Fruits were bought in the market, preferably from organic agriculture, and produced in Portugal. Samples were thoroughly washed with running tap water before use. Fruits were freeze-dried with skin and seeds and ground to a fine powder. The fruit media consisted of 1% (w/v) agar, 10% (w/v) freeze-dried fruit and 0.15% (v/v) orthophosphoric acid in distilled water. Flies were allowed to feed and oviposit on the fruit media for 72 h in an incubator at 24 ± 1 °C, 60 ± 10% RH, and under a photoperiod of 16:8 (L:D) h. Flies were surface washed in sterilized 1X phosphate buffer solution (PBS) pH 7.4 and stored at − 80 °C until use. Five vials per host were used, and the experiment was repeated in three different weeks under the same conditions (three temporal independent experiments).

### Fruit media total soluble sugars and protein quantification

Total soluble sugars (TSS) of the different fruit media were quantified, following the anthrone method described by Dias et al.^52^. Briefly, 50 mg of each fruit medium (n = 3) were homogenized in 1 mL EtOH 80% in a FischerBrand™ BeadMill 24 Homogenizer with one 2.8 mm ceramic bead at 3 m/s for 60 s (ThermoFischer Scientific, Massachusetts, USA). The homogenate was transferred to a 15 mL tube with a final volume of 10 mL EtOH. After 1 h in a water bath at 80 °C and centrifugation at 10,000×*g* for 10 min in a pre-chilled centrifuge (4 °C) (Mega Star 1.6R, VWR, Pennsylvania, USA), TSS were quantified in a microplate spectrophotometer (FLUOstar Omega, BMG LABTECH, Ortenberg, Germany). Absorbance was read at 625 nm, and TSS levels were calculated based on a glucose standard curve (0–1.2 mg/mL) and presented as µg/mg media.

For the quantification of fruit medium protein content, total proteins were extracted according to the salt/alkaline extraction method described by Maehre et al.^53^, with some modifications. Firstly, 50 mg of fruit media was homogenized in 1 mL of salt/alkaline solution (0.1 M NaOH in 3.5% NaCl) in a FischerBrand™ BeadMill 24 Homogenizer with one 2.8 mm ceramic bead at 4 m/s for 60 s (ThermoFischer Scientific, Massachusetts, USA). Samples were incubated for 90 min at 60 °C and centrifuged at 12,000×*g* for 30 min at 4 °C in a pre-chilled centrifuge (Micro Star 17R, VWR, Pennsylvania, USA). An aliquot of 100 µL of the supernatant was used to quantify total soluble protein content with 150 µL of Bradford reagent. After 5 min of incubation at room temperature, absorbance was read at 595 nm in a microplate spectrophotometer (FLUOstar Omega, BMG LABTECH, Ortenberg, Germany). Protein content was calculated based on bovine serum albumin (BSA) standard curve (0–25 µg/mL) and results were expressed as µg/mg media.

### Adult lipids and carbohydrates quantification

Lipids and carbohydrates were quantified enzymatically according to Tennessen et al.^54^. For each assay, males and females were individually analyzed, and two flies from the respective sex, corresponding to each experimental week, were used (n = 6 for each sex).

For lipid content (TAG), flies were homogenized in 100 µL of cold PBST (PBS 1X, pH = 7.4, 0.05% Tween 20) in a FischerBrand™ BeadMill 24 Homogenizer with one 2.8 mm ceramic bead at 2.4 m/s for 60 s (ThermoFischer Scientific, Massachusetts, USA). 10 µL of the homogenate were transferred to a new microtube and stored at − 80 °C for protein quantification. The remaining homogenate was incubated at 70 °C for 10 min. From each sample, 20 µL were transferred to a set of two microtubes; to one set, 20 µL of PBST were added to measure free glycerol, and 20 µL of triglyceride reagent (Sigma-Aldrich, Missouri, USA) were added to the other set of microtubes to measure total glycerol. Samples were incubated at 37 °C for 60 s and centrifuged at full speed for 3 min. Then, 30 µL of each sample were transferred to a 96-well plate, and 100 µL of free glycerol reagent (Sigma-Aldrich, Missouri, USA) were added to each sample. After incubation at 37 °C for 5 min, absorbance was measured at 340 nm in a microplate spectrophotometer (FLUOstar Omega, BMG LABTECH, Ortenberg, Germany). TAG equivalent levels were quantified based on a glycerol standard curve (0–1 mg/mL) (Sigma-Aldrich, Missouri, USA) and normalized to the protein content of each fly.

Glycogen and glucose levels were quantified with hexokinase (HK) reagent (Sigma-Aldrich, Missouri, USA). Flies were homogenized in 100 µL of cold PBS pH 7.4 in a FischerBrand™ BeadMill 24 Homogenizer with one 2.8 mm ceramic bead at 2.4 m/s for 60 s (ThermoFischer Scientific, Massachusetts, USA). An aliquot of 10 µL of the homogenate was transferred to a new microtube and stored at − 80 °C for protein quantification. Samples were incubated at 70 °C for 10 min and centrifuged at maximum speed for 3 min in a pre-chilled centrifuge (Micro Star 17R, VWR, Pennsylvania, USA). After diluting the supernatant (1:3), 20 µL were added to each of two microtubes; 20 µL of amyloglucosidase (AS) were added to one set of microtubes to measure glycogen and 20 µL of PBS were added to the other set to measure free glucose. After incubation, at 37 °C for 1 h, 30 µL were transferred to a 96-well plate and 100 µL of HK reagent were added to each well. Samples were incubated at room temperature for 15 min and the absorbance was measured at 340 nm in a microplate spectrophotometer (FLUOstar Omega, BMG LABTECH, Ortenberg, Germany). Glucose levels were quantified based on a glucose standard curve (0–1.6 mg/mL) (Sigma-Aldrich, Missouri, USA). For glycogen, the absorbance of the PBS-treated sample was subtracted from the sample treated with AS, and the levels were calculated based on a glycogen standard curve (0–1.6 mg/mL) (Acros Organics, ThermoFischer Scientific, Massachusetts, USA). Results were normalized to the soluble protein content of each fly.

To measure soluble protein content, the supernatant was diluted two times in PBS pH 7.4. An aliquot of 5 µL of supernatant was transferred to a 96-well microplate and 150 µL of Bradford reagent was added to each well. After incubation at room temperature for 5 min, absorbance was measured at 595 nm in a microplate spectrophotometer (FLUOstar Omega, BMG LABTECH, Ortenberg, Germany). Protein content was calculated based on a BSA standard curve (0–2 mg/mL) (Sigma-Aldrich, Missouri, USA).

### RNA isolation

Total RNA from a pool of 2 randomly chosen male and female flies fed on each host from each temporal replicate (n = 3 pools of 2 adult flies) were isolated separately with a Tri-reagent (NZYol, NZYTech, Lisbon, Portugal) following the manufacturers’ instructions for small amounts of tissue. Flies were homogenized in 800 µL of NZYol in a FischerBrand™ BeadMill 24 with one 2.8 mm ceramic bead at 2.4 m/s for 60 s (ThermoFischer Scientific, Massachusetts, USA). The aqueous phase was transferred to a new microtube and, after adding chloroform, samples were centrifuged at 12,000×*g* for 15 min in a pre-chilled (4 °C) centrifuge (Micro Star 17R, VWR, Pennsylvania, USA). RNA was precipitated with isopropanol for 1 h at − 20 °C. After washing with EtOH 70%, the pellet was air-dried, and then resuspended in 80 µL of DEPC-treated H_2_O. DNAse treatment was applied to all samples with DNAse I according to the manufacturers’ instructions (NZYTech, Lisbon, Portugal). RNA was stored at − 80 °C until use.

### Gene expression

Total RNA was reverse transcribed with the NZY First-Strand cDNA Synthesis Kit (NZYTech, Lisbon, Portugal). Quantitative real-time PCR (RT-qPCR) was performed to quantify the gene expression of genes involved in relevant metabolic pathways. All genes and primer sequences are detailed in Table 2. After an initial denaturation step (2 min at 95 °C), RT-qPCR reactions were run for 45 cycles (5 s at 95 °C and 30 s at 60 °C), with a final extension step at 65 °C for 5 s (CFX 96, Bio-Rad Laboratories, California, USA). The total volume of each reaction was 20 µL, consisting of 2 µL cDNA (60 ng/µL), 0.8 µL forward primer (10 µM), 0.8 µL reverse primer (10 µM), 10 µL NZYSupreme qPCR Green Master Mix (2x) ROX plus (NZYTech, Lisbon, Portugal) and 6.4 µL Nuclease-free H_2_O. A high-resolution melting curve was performed after each qPCR reaction to ensure amplification specificity. Three technical replicates were used for each cDNA. PCR efficiency was extracted with LingRegPCR^55^. Expression levels were calculated according to Taylor et al.^56^. Normalized gene expression (ΔΔCq) of each target gene was calculated using the geometric mean of three housekeeping genes (*Tbp*, *ArgK,* and *Ef1α48D*).

**Table 2:** Primer sequences used for the RT-qPCR gene expression analysis. *Housekeeping genes.

Name	Gene ID	Sequence (5’–3’)	References
TATA binding protein*	*Tbp_F*	CCACGGTGAATCTGTGCT	^57^
	*Tbp_R*	GGAGTCGTCCTCGCTCTT	
Arginine kinase*	*ArgK_F*	CTACCACAACGATGCCAAGA	
	*ArgK_R*	AAGGTCAGGAAGCCGAGA	
Foxo	*Foxo_F*	CTCCCTGAACACGTACAGCA	^58^
	*Foxo_R*	CTTCGACATTGCACTCCAGA	
Elongation factor 1 α*	*Ef1α48D_F*	TGGGCAAGGAAAAGATTCAC	
	*Ef1α48D_R*	CGGCCTTCAACTTATCCAAA	
Thor	*Thor_F*	ATCATCTCGGATCCAATCCA	
	*Thor_R*	ATTTGCGGAAGGGAGTACG	
Yolk protein 1	*Yp1_F*	CATTGAGCGTCTGGAGAACA	
	*Yp1_R*	AGTTCTGCCTCTGCTTCAGG	
Insulin receptor	*InR_F*	GCATCAAACGTGAAAGCGGT	^59^
	*InR_R*	ATCCAGCACATACAACGCGA	
Trehalose-6-phosphate synthase 1	*Tps1_F*	CGAACATTCCGTGTGACATC	This study
	*Tps1_R*	AGGGACATCTGCATCTGGAC	
Adipokinetic hormone	*Akh_F*	GCAAGACCTCCAACGAAATG	
	*Akh_R*	GTGTGCGTGCTAGACATCGT	
Trehalase	*Treh_F*	GATGCAGGCTAAAAACCAGA	
	*Treh_R*	GGGTTCTCCTTCCAATCAGT	
Sorbitol dehydrogenase-2	*Sodh_2_F*	TGGGTCAGTACAACCTTTGC	
	*Sodh_2_R*	CGGCGTGTTTGTAGTACCTC	

### Statistical analysis

All analyses were performed using GraphPad Prism 9 software (GraphPad Software, California, USA). Host media TSS were analyzed with a One-Way ANOVA followed by a Tukey test for multiple comparisons. Lipids, carbohydrates, and gene expression differences among male or female flies were analyzed with a Two-Way ANOVA followed by a Tukey test for multiple comparisons; differences between male and female flies were assessed with a Two-Way ANOVA followed by a Šídák's multiple comparisons test. Statistically significant differences were considered when p ≤ 0.05. All values are presented as mean ± SEM. PCA analysis was performed with the mean of each analysis’s parameter for males and females, separately.

### Ethics statement

All fruits used in this study for the feeding assays were bought in a local supermarket. The infested raspberries which allowed the establishment of the SWD colony were kindly provided by Cristiana Correia from her family’s raspberry trees.

## Data Availability

The gene expression data analysed during the current study are available in the figshare repository (https://doi.org/10.6084/m9.figshare.20359758).
